# Prediction of Moisture Content for Congou Black Tea Withering Leaves Using Image Features and Nonlinear Method

**DOI:** 10.1038/s41598-018-26165-2

**Published:** 2018-05-18

**Authors:** Gaozhen Liang, Chunwang Dong, Bin Hu, Hongkai Zhu, Haibo Yuan, Yongwen Jiang, Guoshuang Hao

**Affiliations:** 1 Tea Research Institute, The Chinese Academy of Agricultural Sciences, Hangzhou, 310008 China; 2 College of Mechanical and Electrical Engineering, Shihezi University, Shihezi, 832003 China; 3 Department of Food Science, University of Copenhagen, Frederiksberg, 999017 Denmark; 4 Jiande Municipal Bureau of Agriculture, Hangzhou, 311600 China

## Abstract

Withering is the first step in the processing of congou black tea. With respect to the deficiency of traditional water content detection methods, a machine vision based NDT (Non Destructive Testing) method was established to detect the moisture content of withered leaves. First, according to the time sequences using computer visual system collected visible light images of tea leaf surfaces, and color and texture characteristics are extracted through the spatial changes of colors. Then quantitative prediction models for moisture content detection of withered tea leaves was established through linear PLS (Partial Least Squares) and non-linear SVM (Support Vector Machine). The results showed correlation coefficients higher than 0.8 between the water contents and green component mean value (G), lightness component mean value (L^*^) and uniformity (U), which means that the extracted characteristics have great potential to predict the water contents. The performance parameters as correlation coefficient of prediction set (Rp), root-mean-square error of prediction (RMSEP), and relative standard deviation (RPD) of the SVM prediction model are 0.9314, 0.0411 and 1.8004, respectively. The non-linear modeling method can better describe the quantitative analytical relations between the image and water content. With superior generalization and robustness, the method would provide a new train of thought and theoretical basis for the online water content monitoring technology of automated production of black tea.

## Introduction

Withering is a key step in the processing of congou black tea, which directly determines the quality of subsequent steps and the flavoring features of black tea^1,2^. During the process of withering, a series of physical and chemical changes take place when the moisture content in the fresh tea leaves disappears gradually^3^. When the stems and leaves are withering, the luster on the leaf surface is fading away, the color of leaves is turning to dark green, and the grass flavor is vanishing^4^. The moisture loss rate of fresh leaves is usually taken as the quantitative indicator to determine a proper withering^5,6^. When the moisture content is about 58~62%^7^, the withering is considered as a proper withering. In actual production, the fresh tea leaves as well as their physical features are usually judged by sensory experience, which often takes “twists and turns continuously, soft when pinching with fingers, squeeze the withered leaves and loose hand and the leaves can stretch slowly” as the judgment standard of proper withering^8^. However, this method is easily affected by light, experience, subjective judgments, visual and tactual factors, etc. Besides, people may distinguish the small differences between similar colors or appearances, but they can hardly identify the specific levels. As human’s descriptions on color and texture are qualitative^9^, it is hard to realize an accurate, standard and objective evaluation on the moisture content during the withering process. In addition, traditional moisture content measuring method is costly and time-consuming, and may damage the samples and cannot be monitored in real-time^10^.

Machine vision is an instrument detection method using computer, camera, and other related equipment to identify, track, and measure the target; it also includes graphic processing^11^. It can be used for on-line, rapid and non-destructive testing of texture characteristics such as shape, size, color and texture of foods and agricultural products. The machine vision system can be used to obtain the images of tea leaves, and then to extract tea leaves’ color and texture features. In this way, the surface features of tea leaves can be quantified and accurately described. The feasibility of this method for tea quality evaluation has been proved by practical applications^12^. Based on the image RGB histogram features, Borah S. *et al*.^13^ establish a qualitative assessment method for CTC black tea fermentation appropriateness. Yan Jun^14^ presents a qualitative description method according to tea samples’ external feature parameters using the standard samples of roasted green tea as study objects. Yudong Zhang, Wang shuihua *et al*.^15–17^ designed an automatic tea-category identification (TCI) system to Identification of Green, Oolong and Black Teas, which used a 3-CCD digital camera, and then extracted 64 color histogram features and 16 wavelet packet entropy (WPE) features to obtain color information and texture information, respectively. He Yong, Li Xiaoli *et al*.^18,19^ used multispectral imager to acquire the tea leaf images under certain wavelength; with this method, the shape features and gray-level co-occurrence matrix texture features were obtained, which were then used to determine green tea’s classifications. Tang Zhe *et al*.^20^ presented an identification method for the tenderness of fresh tea leaves using texture features and support vector machine.

However, machine vision is rarely used in the research of black tea’s withering process and moisture detection^3,8,12^. Gejima Y. N. *et al*.^21^ used vision technology to monitor the color changes during the de-enzyme process, and to establish a model of image color features and moisture loss rate. Li Jie *et al*.^22^ made an image acquisition device with camera and bellows; they also used Photoshop filter fuzzy algorithm to establish a new method of fetching unbroken tea color with computer, named L Method, extract the mean values of the L^*^, a^*^, and b^*^ color channel from the images of fresh tea leaves; in this way, they obtained the color changes along with the withering level. Scanned with resolution 100 dpi, the credible interval (95%) of the result is: L^*^ = 0, −0.45 ≤ a^*^ ≤ 0.45, −0.71 ≤ b^*^ ≤ 0.71. When the scanned area is 11 cm × 11 cm or more, the color parameters of tea scanning photo may fully stand for the tea actual color. The SE is: −0.16 ≤ L^*^ ≤ 0.16, −0.13 ≤ a^*^ ≤ 0.13, b^*^ = 0. Hua, Jiang *et al*.^23^ studied the color aberration (L^*^, a^*^, b^*^ value) changes during black tea’s withering process under different ambient humidity conditions.

The changes of fresh tea leaves’ external shape characteristics during the withering process can be classified into three parts: color change, the surface smoothness and shrinking degree of the tea leaves. The current researches are focusing on tea leaves’ surface color changes during withering, which ignore the external shape texture features caused by moisture loss. But texture features are important indicators of tea leaves’ shape changes, such as the texture structure wrinkle and shrink. Besides, the correlation between image features and moisture loss rate, and the quantitative analytical relation are still undefined. The aim of this research is to realize quick and nondestructive examination of moisture content during tea processing. To achieve this goal, image acquisition system is applied in this research to obtain the visible light image of withering leaves, and to extract the texture and color features; linear method of PLS and BN and nonlinear method of BP-ANN, SVM and RF are used for the establishment of moisture quantitative characterization model for withering tea leaves. In this research, the performances of models are analyzed through comparison to explore the relations between color, texture features, and moisture. Finally, a new evaluation model with high precision and strong generalization is obtained. This research can provide theoretical support for the feedback control technology and the online moisture testing during the automated production of black tea.

## Results and Analysis

### Correlation analysis on visual features and moisture content

A correlation analysis on the sample moisture content and image feature variables was conducted. The results are shown as Table 1. Except blue component mean value (B) and hue mean value (H) variables (bold in Table 1), all the image indicators were significantly correlated with moisture content (p < 0.01). Moisture content was significantly positively correlated with red component mean value (R), green component mean value (G), saturation mean value (S), visible light mean value (V), b component mean value (b^*^), lightness component mean value (L^*^) and significantly negatively correlated with a component mean value (a^*^). It indicated that, the higher the moisture content, the brighter and greener the leaves, the more intense the color. During the correlation analysis on texture and moisture content, except third moment (μ_3_) value, all the feature variables were significantly correlated with moisture content (uniformity (U) is significantly negatively correlated), which showed that the surface morphology and structure of the leaves changed greatly during the withering process.

**Table 1:** Correlation between image feature values and moisture content.

parameter	Y	R	G	B	H	S	V	a^*^	b^*^	L^*^
R	0.709^**^	1	0.866^**^	0.591^**^	−0.286	−0.158	0.987^**^	0.104	−0.080	0.939^**^
G	0.856^**^	0.866^**^	1	0.141	0.186	0.336	0.807^**^	−0.402	0.411	0.984^**^
B	**−0.051**	0.591^**^	0.141	1	−0.690^**^	−0.884^**^	0.696^**^	0.836^**^	−0.843^**^	0.310
H	**0.350**	−0.286	0.186	−0.690^**^	1	0.756^**^	−0.314	−0.827^**^	0.755^**^	0.037
S	0.490^*^	−0.158	0.336	−0.884^**^	0.756^**^	1	−0.284	−0.988^**^	0.996^**^	0.168
V	0.641^**^	0.987^**^	0.807^**^	0.696^**^	−0.314	−0.284	1	0.215	−0.206	0.897^**^
a^*^	−0.560^**^	0.104	−0.402	0.836^**^	−0.827^**^	−0.988^**^	0.215	1	−0.993^**^	−0.236
b^*^	0.559^**^	−0.080	0.411	−0.843^**^	0.755^**^	0.996^**^	−0.206	−0.993^**^	1	0.247
L^*^	0.860^**^	0.939^**^	0.984^**^	0.310	0.037	0.168	0.897^**^	−0.236	0.247	1
m	0.641^**^	0.987^**^	0.807^**^	0.696^**^	−0.314	−0.284	1.000^**^	0.215	−0.206	0.897^**^
δ	0.676^**^	0.177	0.620^**^	−0.656^**^	0.697^**^	0.917^**^	0.057	−0.929^**^	0.937^**^	0.478^*^
r	0.619^**^	0.063	0.532^**^	−0.744^**^	0.737^**^	0.960^**^	−0.060	−0.965^**^	0.972^**^	0.378
μ_3_	**0.246**	0.495^*^	0.416^*^	0.068	−0.414^*^	0.115	0.389	−0.028	0.128	0.431^*^
U	−0.840^**^	−0.517^*^	−0.859^**^	0.322	−0.553^**^	−0.712^**^	−0.424^*^	0.763^**^	−0.763^**^	−0.765^**^
e	0.837^**^	0.423^*^	0.806^**^	−0.421^*^	0.631^**^	0.783^**^	0.325	−0.829^**^	0.827^**^	0.695^**^

Note: ^**^Correlation is significant at the 0.01 level, ^*^Correlation is significant at the 0.05 level.

In addition, the image feature variables were also correlated with each other to some extent. For example, the correlation between R and V was as much as 0.987. The correlation between these variables would cause large information overlap and overfitting of the established model, as well as excellent fitting performances of the calibration set but inferior external calibration^24^. Hence, in further modeling, dimensional reduction of principle components should be conducted on image feature variables, which could eliminate collinearity and reduce machine learning time when retaining image information correlated with moisture content.

### Analysis on changes of color and texture features

**Figure 1:**
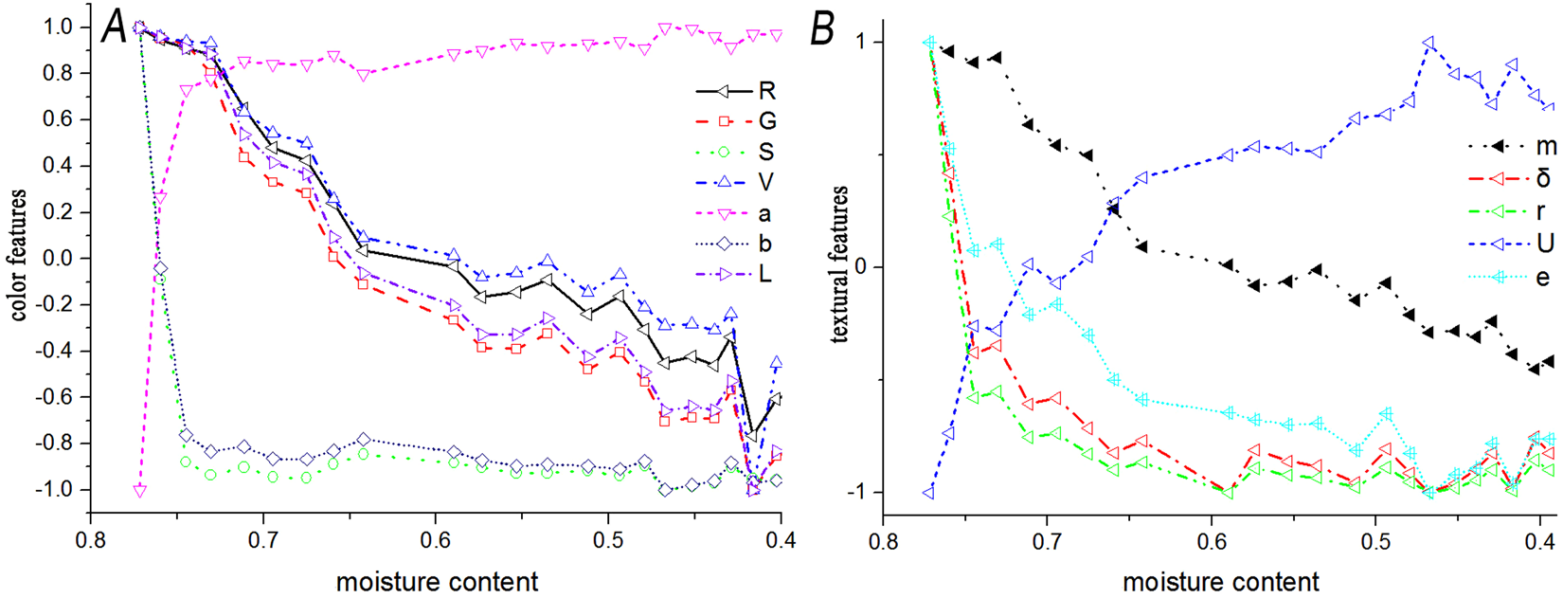
The changes of color (**A**) and texture (**B**) features with the different moisture content.

Generally, the color and texture feature variables extracted were multivariate high-dimensional arrays with different dimensions and orders of magnitude. Hence, in order to investigate the dynamic change rules of image feature variables (significantly correlated with moisture content) during the withering process, this study first conducted Zscore on original data to eliminate the limitation of dimension and order of magnitude. The results were shown as Fig. 1. As shown in Fig. 1(A), with the decrease of moisture content, R, G, V, L^*^ presented a linear decrease, a^*^ presented an upward trend of fast-to-slow, S and b^*^ presented a trend of rapid decrease to slow decrease, U of texture features presented a gradual increase, average gray value (m), standard deviation (δ), smoothness (r), and entropy (e) presented a rapid decrease and then a slow decrease after the moisture content reduced to 60%.

### PLS linear correlation model

**Figure 2:**
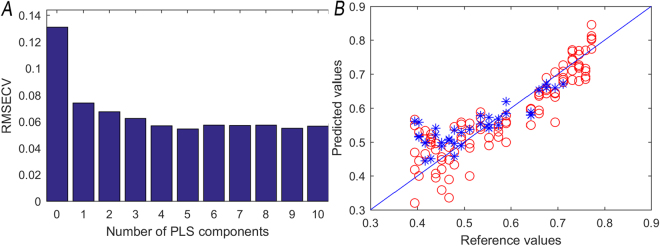
Principal component selection (**A**) and scatter plot of prediction set (**B**).

A correlation model between image features and moisture content was proposed during the withering period of black tea in order to realize a rapid and nondestructive inspection of moisture content. As a widely used typical mathematical modeling method, PLS could effectively solve the problem of multi-collinearity and raise the accuracy of the model^17^. Figure 2A showed the corresponding RMSEC values of PLS linear models established with different NPC (number of principle components). Figure 2B showed the relations between the predicted values and the measured values. The corresponding NPC of the minimal RMSEC(0.082) was 5; the correlation coefficient of the prediction set (R), root mean square prediction error (RMSEP), Bias, standard deviation (SEP), coefficient of variation (CV) and relative percent deviation (RPD) were 0.8349, 0.0607, 0.0262, 0.0073, 0.1086 and 0.9834; the relations between the predicted value and measured value was shown as Fig. 2B.

### Nonlinear correlation model of SVM

Although typical neural network method could deal with the problem of nonlinearity, its randomness and overfitting of the initial values has limited its use. Raised by Vapnik, SVM was a high dimensional information processing tool and a more effective multivariate modeling analytical method with great potentiality. SVM replaces the inner product operation in high-dimensional space by introducing a kernel function and solves the nonlinear fitting problem. Therefore, the selection of the kernel function directly affects the generalization ability of the model. SVM is established on the Li-svmlab toolbox of Matlab^25^, 10 groups of principle components as the input, and moisture content of the withered leaves as the output of the network. Four kinds of kernel functions such as linear, polynomial, Radial Basis Function (RBF), and sigmoid are selected respectively to establish the prediction model of the water content of the withering leaf, and the optimal kernel function was determined by comparison. From the Table S1, the SVM model based on the RBF kernel function has the best predictive performance, and its RPD value is 1.7562, which is higher than the SVM model of other kernel functions.

**Figure 3:**
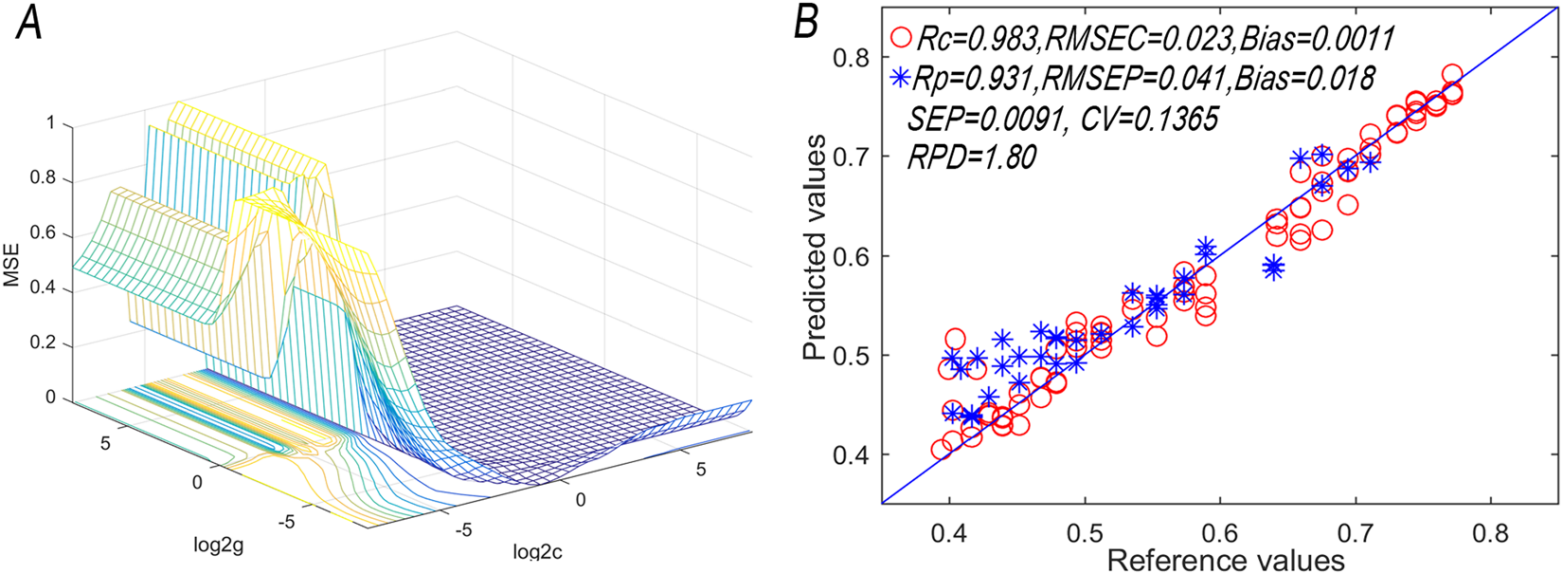
Model parameters optimization (**A**) and scatter plot of prediction set (**B**).

When SVM chose RBF as its kernel function, considering the influences brought by penalty parameters (c) and kernel function parameters (g) to the modeling results^26^, grid searching technique and cross validation were used to conduct a global optimization on c and g. The optimization process was shown as Fig. 3A. As shown, when c = 0.32988 and g = 0.0089742, the RMSEC of the model has the minimal value of 0.0239, and the Rp, RMSEP, Bias, SEP, CV and RPD of the prediction set are 0.9314, 0.0411, 0.0185, 0.0091, 0.1365 and 1.8004 respectively. The relations between the prediction value and measured value was shown as Fig. 3B.

### Nonlinear model of Random Fores

Random Forest (RF) is a decision tree-based integration algorithm whose basic unit is a decision tree. When the RF model is built, the number of inputting principal components (PCs) and the number of decision trees (N) directly affect the accuracy of the model, so the PCs and N need to be further optimized (within a certain range of parameters). Twenty N (50 to 1000 in steps of 50) and 10 PCs (1 to 10 in steps of 1) were selected, then the parameters were optimized using the RMSEC values of the model.

The optimization result was shown in Fig. S1. As shown in Fig. S1(A), when PCs = 4, N = 350, the RMSEC of the model has the minimal value of 0.0012 in the TFs prediction model, and Rp, RMSEP, Bias, SEP, CV, and RPD of the prediction set were 0.891, 0.058, −0.007, 0.011, 0.190 and 1.612, respectively. The relations between prediction value and measured value was shown as Fig. S1(B).

### Model comparison and discussion

**Figure 4:**
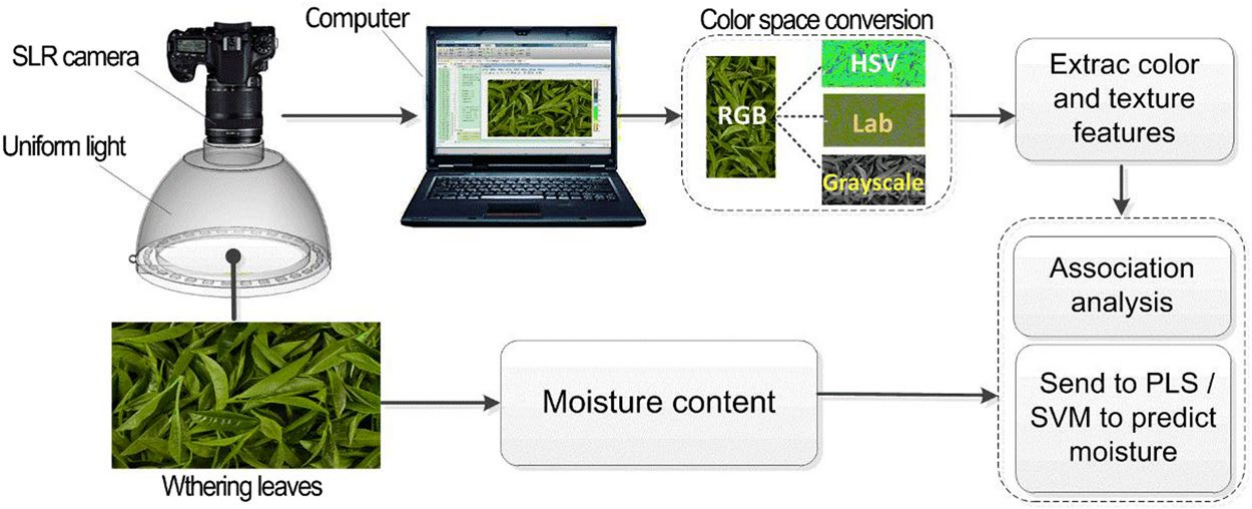
Flowchart of the algorithm employed for color and texture measurement.

In this part, the performances of linear model (PLS and BN) and nonlinear models (BP-ANN, SVM and RF) were compared (Table 2). The results showed that the Rp, RMSEP and Bias of the prediction set of the nonlinear model were obviously better than that of linear model. SVM model has the best prediction performance with RPD > 1.8, which could be used for quantitative analysis of moisture content. The small SEP and CV showed that the model has a small degree of sample deviation and discrete variation with preferable accuracy and prediction capability. In addition, according to Figs 3B and 4B, the prediction scatter distribution of PLS model is rather dispersive, which indicated that the linear regression model is weak in generalization due to the overfitting and couldn’t make effective prediction on external independent samples. Compared with PLS model, the prediction scatter of SVM model was more converged to slope.

**Table 2:** Results of different models for each prediction of moisture.

Methods	NPC	Calibration set			Prediction set					
		Rc	RMSEC	Bias	Rp	RMSEP	Bias	SEP	CV	RPD
PLS	5	0.8983	0.0821	0.0417	0.8349	0.0607	0.0262	0.0073	0.1086	0.9834
BN	5	0.9644	0.0352	0.0035	0.5968	0.0918	0.0005	0.0169	0.2026	1.1743
BP-ANN	4	0.9522	0.0364	0.0117	0.8949	0.0513	0.0198	0.0093	0.1381	1.6205
SVM	4	0.9838	0.0239	0.0011	0.9314	0.0411	0.0185	0.0091	0.1365	1.8004
RF	4	0.9858	0.0464	0.0003	0.9172	0.0472	0.0240	0.0121	0.1390	1.6379

BN, Bayesian Network; SD, standard deviation; NPC, used latent variables; RMSEC, root mean square error of calibration; RMSEP: root mean square error of prediction; SEP, standard error of prediction; CV, coefficient of variation; RPD, residual predictive deviation value of prediction.

With the decrease of moisture content during the withering process, the chemical components existed in the fresh leaves are also changing. For example, polyphenols were degraded, with the total amount reduced and oxidation products continuously increased with the strengthening of the withering^3,6^; under the effect of chlorophyllase, chlorophylls were hydrolyzed to chlorophyllide (Cda, Cdb) and phytol, which induced the changes of colors of tea leaves^2^. Meanwhile, the constantly losing moisture content of the fresh leaves may cause cell collapse and structural damages, and further lead to morphological changes and shrinkage of the leaves^8,23^. Due to the complicated influences from moisture, biochemical components and physical properties, there were certain nonlinear factors of the image features. PLS method only dealt with the linear relations between variables and results, and ignored the potential nonlinear factors^27,28^. Hence, compared with PLS model, nonlinear methods such as BP-ANN and SVM were featured with better generalization capability and prediction accuracy^17^.

## Materials and Methods

### Sample collection

In this research, newly picked fresh tea leaves were collected as experimental materials; the variety of the tea leaf was Jiukeng; the tenderness was 1 bud and 2 leaves. In April 2017 the experiment was conducted at Jiande Qiandao Yinzhen Tea Company. The fresh tea leaves were placed in an artificial climate box for withering; the ambient temperature was set to be 35 °C; the relative humidity was 50%. Samples were taken every 0.5 h during the withering process; for each sampling, 6 groups of samples were collected for image acquisition. The mass of each group was 15 ± 0.5 g which was uniformly tiled in a sample cell of size Φ70 mm, and the sample cell is placed under a uniform light source for image acquisition. Take 3 g from the tea sample after the image collected, and test the moisture content by a moisture analyzer (MA35M-000230V1, Sartorius). The test is repeated 3 times, and the average value of 3 times is taken as the moisture value of the collected image. After 11 hours of withering, the moisture content dropped from the original 77.18% to 39.53%, representing the completion of withering process. Totally, 138 sample images were collected based on 23 time points. The moisture contents of 138 samples were used as the reference values of modeling. 95 effective samples were selected as the training set in accordance with the Mahalanobis distance-based Kennard-Stone (KS) algorithm^29^; the remaining 43 samples were used as the external validation set (prediction set)^30^.

### Machine vision system

**Figure 5:**
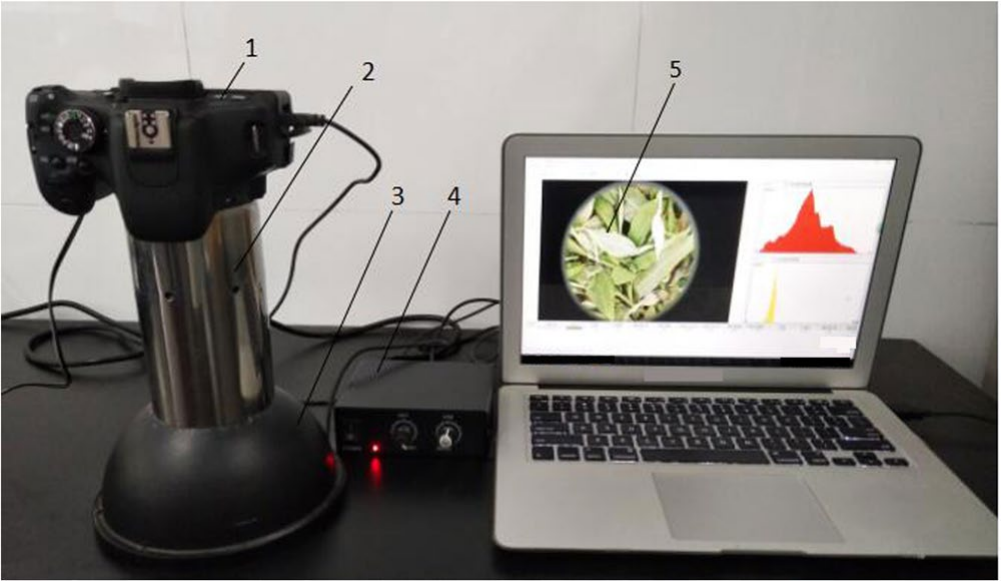
Computer image acquisition system physical drawing (1. Canon DS60D, Japan, 18MP; 2. Roller -distance; 3. Cambered uniform light; 4. Controller of Cambered uniform light; 5. Image processing system).

A computer image acquisition system was designed, which consisted of image sensor, sample pool, uniform light, and image software processing system. Image acquisition and data analysis were conducted in line with the technological path in Fig. 4. SLR camera^31^ (Canon DS60D, Japan, 18MP) was used as the image sensor: ISO sensitivity was set to 100; image size was set to 3456 × 2304 pixels; Aperture AV. was set to f/4.0; Exposure time AV. was set to 1/30 s. Cambered uniform light with an intensity of 100 lx was selected as the light source. Images were stored in RAW format. DCRaw v 9.17 was used for software decoding with the following options: the white balance was the same as that of photographing; AHD (Adaptive Homogeneity-Directed Demosaic algorithm) was adopted to remove mosaic^32^, RGB values were transformed into sRGB color space and then stored in 16 digits TIFF format. Image processing system (software copyright number of china: 2013SR122183; 2014SR149549) was developed based on Matlab GUI module; GUI program could automatically analyze the color and texture features of the image. Figure 5. showed computer image acquisition system physical drawing.

The process of image acquisition is as follows: When the tea samples were placed under the cambered uniform light, the parameters of the SLR camera were adjusted. Then the images which were acquired by SLR camera were imported into the image processing system, and the software system automatically divided the 2000 × 1000 pixels area and analyzed the color and texture features of images.

### Image acquisition and feature extraction

When collecting sample image acquisition, a region in size of 2000 × 1000 pixels was automatically segmented from the image by software system. The image feature mean values (color and texture) of this “region of interest” were then extracted. Through the color model conversion between RGB, HSV and CIE Lab^33^, nine color indicators were extracted respectively as R, G, B, H, V, S, L^*^, a^*^ and b^*^^34^.

Based on the statistic gray co-occurrence matrix^35^, a texture analysis was carried out for the images of withering leaves with different moisture contents. In this step, 6 texture features were obtained, including m, δ, r, μ_3_, U, and e; totally, 15 image feature variables were obtained^18,19,36,37^.

### Data processing and analysis

Least square method (PLS) and support vector machine (BP-ANN and SVM) were used for linear and non-linear quantitative model^19,38–41^, respectively.

Least square method (PLS) was a classical multivariate correction method that finds the best function match of a set of data by minimizing the sum of square s of errors. In the process of establishing the PLS model, the best principal component number was determined by root mean square error of calibration in the calibration set, and the number of principal component of the minimum RMSEC value used by the corresponding model was the number of the best principal component factor.

SVM was a learning system using a linear function to assume a space in a high-dimensional feature space. through a non-linear mapping the data samples were mapped to high-dimensional feature spaces and perform linear regression in this space.

Zscore(standardized conversion) method^42^ was adopted to preprocess the original data. Principal component analysis (PCA) was used to extract feature variables from the original data^43^; the extracted data were then used as the input variables of the model. The parameters of Rc, Rp, RMSEC, RMSEP, Bias, RPD, SEP and CV applied in Literature^44–46^ were used as the evaluation indicators for the model performance. Usually, the higher the Rp and RPD, the smaller the RMSEP, SEP, CV and Bias, and the higher the prediction performance and accuracy of the model will be^33^.

## Conclusion

1. Based on linear method of PLS and BN and nonlinear method of BP-ANN, SVM and RF, different models based on machine vision for predicting the moisture content during the withering process of tea leaves were established respectively. The results showed that SVM model has better performance than other model, with correlation coefficient between the prediction value and measured value 0.9314, prediction root-mean-square error 0.0411 and RPD > 1.5. This showed that there exist nonlinear relations between moisture content and image features, and nonlinear method could better represent the quantitative analytical relations between image information and moisture content.
2. The significant correlation has been explicated between the moisture content of withered leaves and the multiple color and texture feature variables. The correlation coefficients of parameters G, L and U are more than 0.8, which showed that leaf image features can be used to represent moisture content changes.
3. The study has provided theoretical basis for the development of moisture online monitoring technology and equipment used in automated withering process of Congo black tea. It has a good application prospect, which could be also applied to the processing of other tea species such as the spreading of green tea and withering of oolong tea.

## Electronic supplementary material

Supplementary Information
