# Supplementary material for: Prediction of Moisture Content for Congou Black Tea Withering Leaves Using Image Features and Nonlinear Method
Source: Sci Rep. 2018 May 18;8:7854. doi: 10.1038/s41598-018-26165-2 (PMC5959864; doi:10.1038/s41598-018-26165-2)
Supplement: Supplementary file 1 — Supplementary Information [file 41598_2018_26165_MOESM1_ESM.docx]

**Supplementary Information**

**Title**

Prediction of Moisture Content for Congou Black Tea Withering Leaves Using Image Features and Nonlinear Method

**Author list and affiliations**

Gaozhen Liang^b^, Chunwang Dong^a*^, Bin Hu^b*^, Hongkai Zhu^c^, Haibo Yuan^a^, Yongwen Jiang ^a^ and *Guoshuang Hao* ^d^

*a. Tea Research Institute, The Chinese Academy of Agricultural Sciences, Hangzhou 310008, China.*

*b.* *College of* *Mechanical and Electrical Engineering, Shihezi University, Shihezi 832003, China.*

*c.* *Department of Food Science,* *University of Copenhagen,* *Frederiksberg 999017, Denmark*

*d.* *Jiande Municipal Bureau of Agriculture; Hangzhou 311600, China.*

*Corresponding author: Chunwang Dong, E-mail addresses:dongchunwang@163.com

** Corresponding author: Bin Hu, E-mail addresses: 2582983022@qq.com.

Tel: +86-571-86653155. Fax: +86-571-86650103.

**Supplementary Table S1**:

**Table.S1** The Influence of kernel function on the performance of SVM model

| **Kernel**  **function** | NPC | **Calibration set** | | **Prediction set** | | |
| --- | --- | --- | --- | --- | --- | --- |
|  |  | Rc | RMSEC | Rp | RMSEP | RPD |
| linear | 10 | 0.8340 | 0.0726 | 0.8239 | 0.0626 | 1.0319 |
| polynomial | 10 | 0.8833 | 0.0647 | 0.6496 | 0.0914 | 0.5386 |
| RBF | 10 | 0.9561 | 0.0385 | 0.9003 | 0.0433 | 1.7562 |
| sigmoid | 10 | 0.8323 | 0.0741 | 0.7567 | 0.0654 | 0.9521 |
| **NPC, used latent variables; Rc ,correlation coefficient of calibration set; RMSEC, root mean square error of calibration; Rp ,correlation coefficient of prediction set; RMSEP: root mean square error of prediction; RPD, residual predictive deviation value of prediction.** | | | | | | |

**Supplementary Figure Legends S1**:

**Fig.S1** Model parameters optimization (A) and scatter plot of prediction set (B)


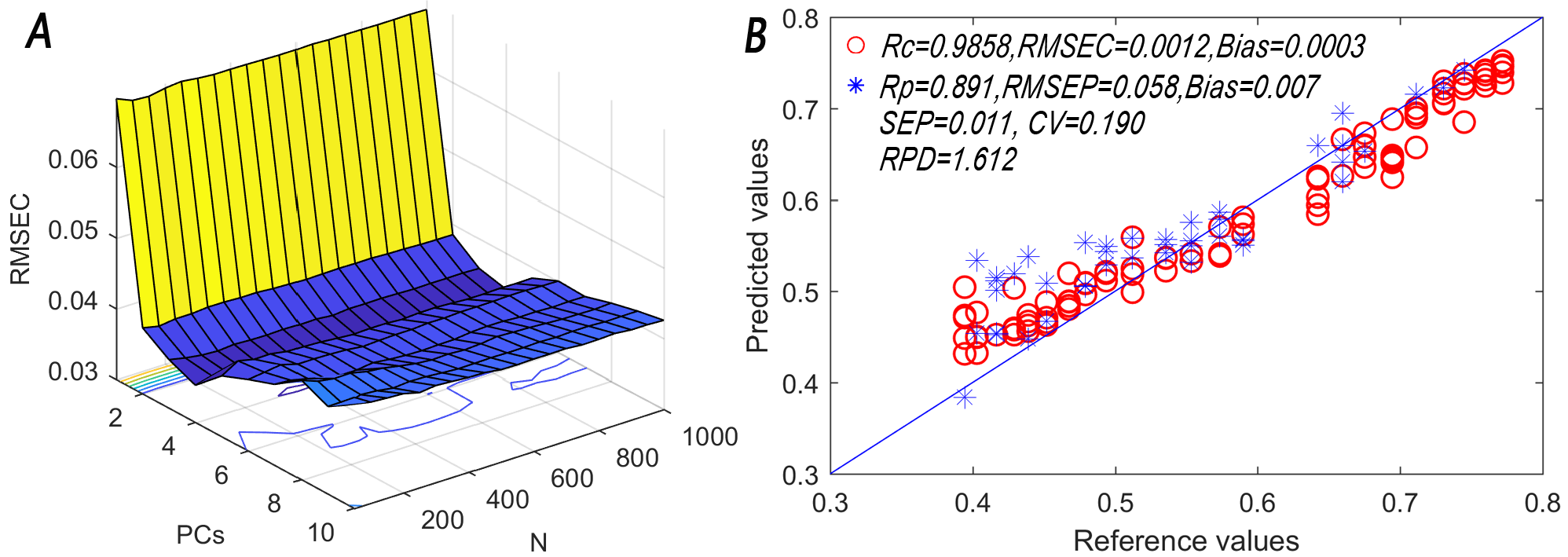


Supplementary Figure S1
